# TRAIL-R2 in the shadows: Epigenetic silencing and clinical implications in breast cancer

**DOI:** 10.18632/oncotarget.28891

**Published:** 2026-06-09

**Authors:** Nuzhat Khursheed, Syed Ishfa Andrabi, Natasha Thakur, Quratul Qadir, Suhail Ashraf, Ambreena Farooq, Safeena Rashid, Farhat Jabeen, Waseen Younis Khan, Allah-u-Deen Khursheed, Mohmmad Afzal Zargar, Asia Asiaf, Showkat Ahmad Ganie

**Affiliations:** ^1^Department of Clinical Biochemistry, University of Kashmir, Srinagar, Jammu and Kashmir 190006, India; ^2^Department of Oncology, Sher-i-Kashmir Institute of Medical Sciences, Srinagar, Jammu and Kashmir 190011, India; ^3^Department of Biochemistry, University of Kashmir, Srinagar, Jammu and Kashmir 190006, India; ^4^Government Medical College Baramulla, Baramulla, Jammu and Kashmir 193101, India; ^5^Department of Biotechnology, Central University of Kashmir, Ganderbal, Jammu and Kashmir 191201, India

**Keywords:** *TRAIL-R2/DR5*, promoter methylation, breast cancer biomarkers, tumor suppressor gene, apoptotic signalling pathways

## Abstract

Breast cancer is considered to be one of the most widespread malignancies, however, its molecular processes are not fully comprehended. Another important epigenetic process that regulates the expression of genes in cancer is promoter methylation. *TRAIL-R2* is also a key mediator of apoptosis but its clinical implications and epigenetic regulation in breast cancer are not clearly understood. In this research, the level of the promoter of the gene *TRAIL-R2* in the matched tumor and normal breast tissues was analyzed using methylation-specific PCR and the level of the mRNA and protein products in the matched tumor and normal breast tissues were measured using quantitative real-time PCR and western blotting. Methylation of *TRAIL-R2* promoter was significantly enhanced in tumor tissues and was negatively correlated with the levels of mRNA and protein. We found that hypermethylation was much more common in invasive ductal carcinoma patients and in patients with a history of use of oral contraceptives. A decreased expression of mRNA of *TRAIL-R2* was significantly related to advanced TNM stage (III–IV) and the absence of progesterone receptor, and low protein expression was significantly related to postmenopausal status. These results suggest that aggressive clinicopathological phenotypes are associated with *TRAIL-R2* silencing via promoter hypermethylation that may be relevant as a prognostic biomarker and therapeutic target in breast cancer.

## INTRODUCTION

According to GLOBOCAN 2022, cancer is the second most common cause of death globally, after cardiovascular illnesses. According to the site of occurrence, breast cancer has the highest incidence and comes in second only to lung cancer in terms of cancer-related death [1]. In India, the leading cause of death for women who are at immediate risk for illness is breast cancer [2]. More individualised therapies are required, rather than generalised ones, to create a more effective cancer treatment strategy. Considering this, searching for more accurate cancer biomarkers is essential to developing a more targeted cancer treatment plan [3]. Programmed cell death, or apoptosis, is a fundamental mechanism for maintaining morphogenetic equilibrium in pathological and early developmental circumstances at any point in a person’s life [4]. Furthermore, during the disease, apoptosis is also a crucial defence mechanism against immunological responses and removing damaged cells [5]. Apoptosis is the initial mechanism that inhibits the growth of tumour cells after cancer has begun [6]. Numerous studies have identified the dysregulation of divergent apoptotic components throughout the initiation and advancement of cancer [7]. Understanding the expression and regulation of these apoptotic factors in carcinogenesis can aid in monitoring disease progression and developing targeted therapies [8]. One of the primary apoptotic pathways is regulated by the tumour necrosis factor (TNF)-related apoptosis-inducing ligand *(TRAIL/Apo2L)*, which binds to four extracellular receptors in the TNF-receptor superfamily: [9, 10], *TRAIL-R1* (Death receptor DR4) [11] and *TRAIL-R2* (KILLER/DR5; Apo2, TRICK2) [12] that contain cytoplasmic Death Domain (DD) and could induce apoptosis upon TRAIL binding [13–15]. On the other hand, the death signal cannot be transduced by *TRAIL-R3* (*DcR1, TRID, LIT, DR1*) [16] with no DD and *TRAIL-R4* (*TRUNDD, DR2*) [17] with a much-shortened DD [18]. Human chromosomes 8p21–p22 are home to all four TRAIL receptors [19]. A protein known as tumour necrosis factor-(TNF)-related apoptosis-inducing ligand *(TRAIL)* has the power to be an anticancer agent by causing apoptosis in a variety of human tumour cells [20]. Since *TRAIL* preferentially triggers apoptosis by interacting with death receptors on tumour cells, a prior study has shown that it is harmless for normal cells [21].

*TRAIL-R2* has been extensively researched in breast cancer because of its dual effects in apoptosis and tumor progression. The activation of *TRAIL-R2* activates apoptosis through the extrinsic pathway, which supports its therapeutic potential [22]. Nevertheless, Oh Y-T et al. [23] have reported that *TRAIL-R2* can equally activate non-apoptotic pathways *(NF-kB*, *MAPK, PI3K/Akt)*, to promote tumor survival and metastasis. Notably, there is variation in *TRAIL-R2* expression in breast cancer, which has been found to influence its functional outcome; higher expression of *TRAIL-R2* may increase the extent of apoptosis, but it has also been associated with aggressive tumor behaviour. In line with this, Fritsche H et al. [24] demonstrated that *TRAIL-R2* induces skeletal metastasis in a breast cancer model. Therefore, *TRAIL-R2* portrays a situational tumor suppressor or promoter, depending on its expression and *in situ* signaling.

Hormonal and reproductive variables significantly influence the risk of breast cancer. Prolonged exposure to both endogenous and exogenous estrogens, including advanced age at menopause and oral contraceptive use, has been associated with an elevated risk of breast cancer [25, 26]. The purpose of our research was to examine the relationship between clinical staging, various clinicopathological markers, and *TRAIL-R2* expression and epigenetic regulation in breast cancer in the Indian population.

## RESULTS

### Downregulated *TRAIL-R2* expression in breast cancer cases

We found *TRAIL-R2* mRNA in samples of breast cancer and nearby healthy tissue. The expression of *TRAIL-R2* was standardized by the quantity of *GAPDH*. In 71.64% (48/67) of breast cancer cases (Table 1), the level of *TRAIL-R2* expression was determined to be lower than normal. In breast tissue with cancer, *TRAIL-R2* had an average expression level of 0.47 ± 0.20 (mean ± SD), while in neighbouring normal tissue, it had an average expression level of 0.87 ± 0.20 (mean ± SD) (*p* < 0.001), as shown in Figure 1A, 1B. A comparison of participants’ clinicopathological data with *TRAIL-R2* mRNA expression revealed a strong connection with breast cancer type, TNM stage, and progesterone receptor. The mRNA expression of TRAIL-R2 exhibited a strong correlation with breast cancer type (*p* = 0.0002), TNM stage (*p* = 0.04), and progesterone receptor status (*p* = 0.002). The invasive ductal carcinoma (0.3 ± 0.1) exhibited reduced expression compared to other tumor types (0.9 ± 0.8). The advanced phases (III and IV; 0.35 ± 0.1) exhibited reduced expression compared to the early stages (I and II; 0.55 ± 0.6). Progesterone receptor positive tumors (0.32 ± 0.1) exhibited reduced expression compared to progesterone receptor negative cancers (0.54 ± 0.5) (Supplementary Table 1).

**Table 1:** Expression levels of TRAIL-R2 mRNA in breast cancer patients

TRAIL-R2 mRNA	Normal	Tumor
Low expression	11 (16.41%)	48 (71.64%)
High expression	56 (83.58%)	19 (28.35%)
Total(*n*)	*n* = 67	*n* = 67

**Figure 1:**
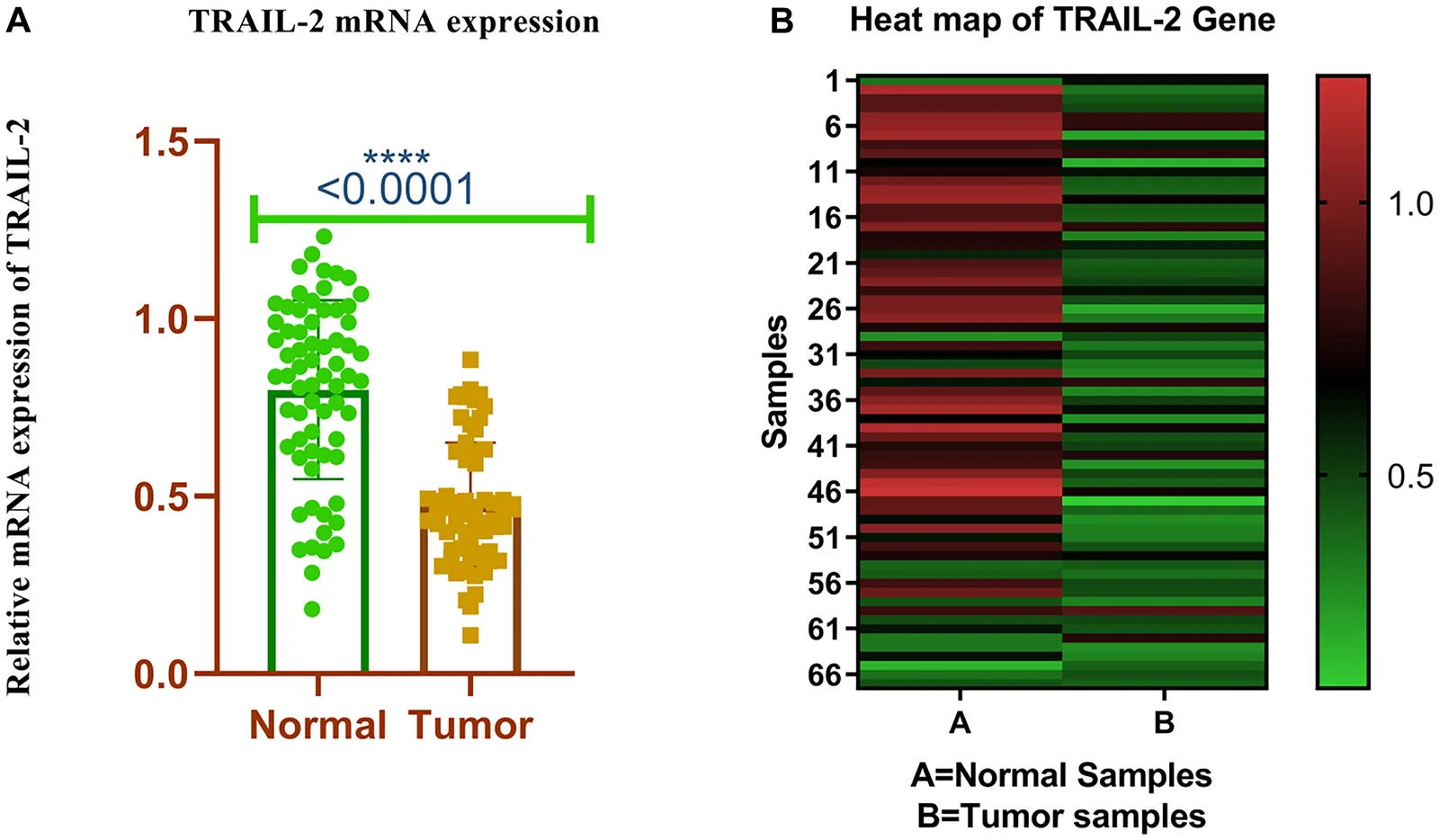
(**A**) Relative mRNA expression of TRAIL-R2 receptor in breast tumor and adjacent normal tissues. (**B**) Heat map plot (analysed by graph pad prism version 8.0.2) of TRAIL-R2 mRNA relative expression (fold change) in breast cancer cases and adjacent normal tissues.

### Low expression of *Trail-R2* protein in breast cancer tissues

In 76.11% (51/67) of the cases tested, *TRAIL-R2* protein expression was determined to be poor. The expression of *TRAIL-R2* protein was standardized by the quantity of beta-actin (Table 2). The expression level of *TRAIL-R2* protein in breast cancer tissues was quantified at 0.25 ± 0.17 (mean ± SD), compared to 0.80 ± 0.47 (mean ± SD) in adjacent normal tissue, exhibiting a statistically significant difference (*p* < 0.001), as depicted in Figure 2B. Figure 2A depicts the representative samples (Patients 1–3) for visualization, however these samples are included in the complete dataset. Figure 2C illustrates the overall quantitative pattern derived from all evaluated samples, including Figure 2A. The expression of the *TRAIL-R2* protein exhibited a significant connection exclusively with menopausal status (*p* ≤ 0.05) (Supplementary Table 2).

**Table 2:** Expression levels of TRAIL-R2 protein in breast cancer patients

TRAIL-R2 Protein	Normal	Tumor
Low expression	13 (19.40%)	51 (76.11%)
High expression	54 (80.59%)	16 (23.88%)
Total(*n*)	*n* = 67	*n* = 67

**Figure 2:**
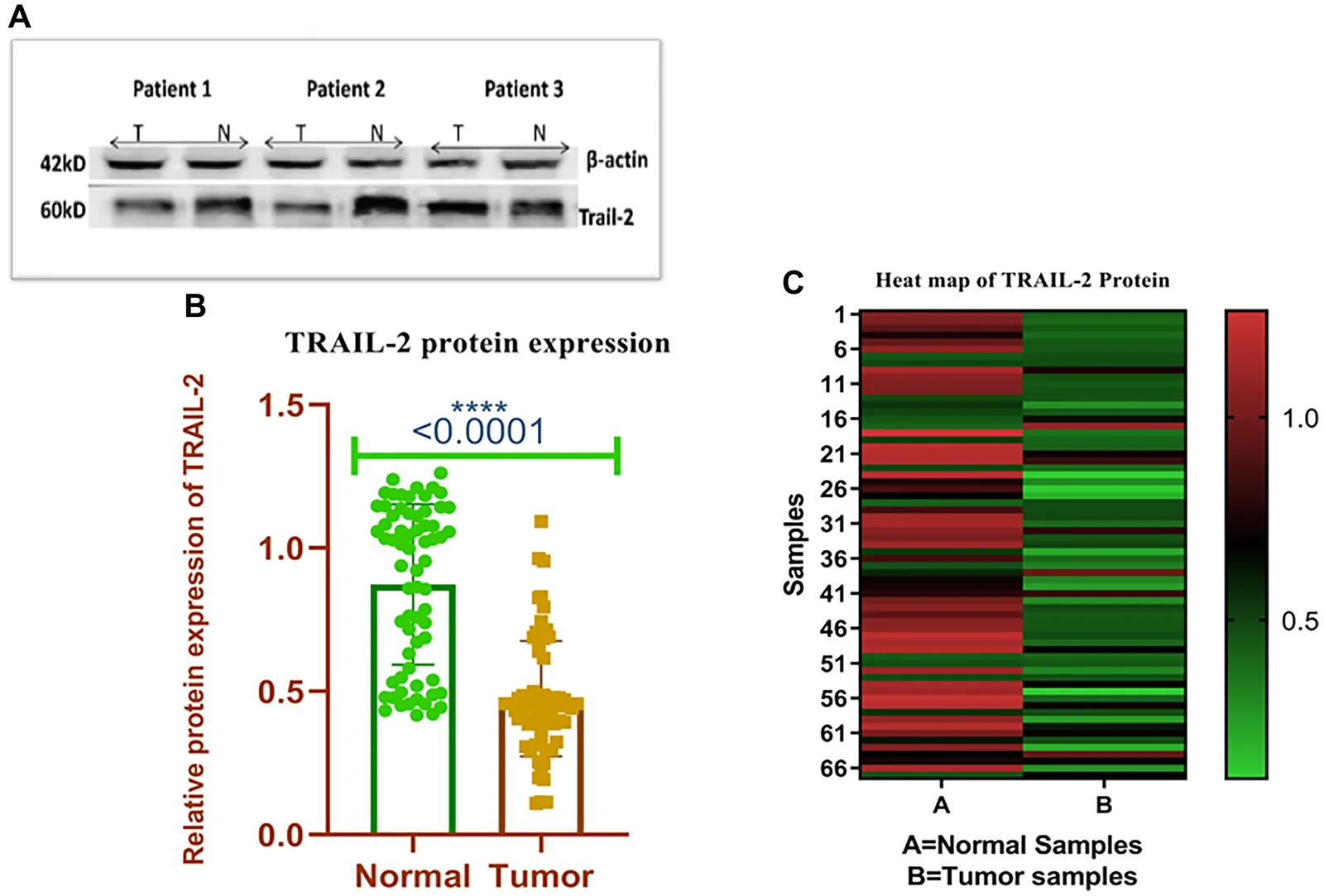
(**A**) The representative western blots show the expression of TRAIL-R2 protein using (Specific antibodies and corresponding loading control β-actin. N represents the protein from the normal site and T represents the protein from tumor site. (**B**) Relative Protein expression of TRAIL-R2 receptor in breast tumor and adjacent normal tissues. (**C**) Heat map plot (analysed by graph pad prism version 8.0.2) of TRAIL-R2 protein relative expression (fold change) in breast cancer cases and adjacent normal tissues.

### Clinicopathological parameters and its correlation with *TRAIL-R2* promoter methylation

Using Methylation Specific PCR, researchers revealed that the *TRAIL-R2* promoter region was methylated in 36 out of 67 (53.73%) breast cancer tissues. In contrast, only 25 out of 67 (37.31%) nearby normal tissues were methylated (Table 3). Figure 3A depicts a standard observation of TRAIL-R2 promoter methylation, while Figure 3B displays the overall distribution of the research cohort. The investigation encompassed all 67 matched tumor tissue and surrounding normal tissue specimens. The proportion of the total number of samples was estimated based on the rates of methylation and unmethylated cases, facilitating a comparison between tumor and nearby normal tissues. A comparison of participants’ clinicopathological data with the methylation state of the *TRAIL-R2* gene indicated a significant correlation between breast cancer subtype and oral contraceptive pill (OCP) usage. Oral contraceptive use (*p* = 0.042) and breast carcinoma type (*p* = 0.05) exhibited positive correlations with TRAIL-R2 promoter methylation. Higher methylation rates were observed in OCP users (13/17) compared to non-users (22/46), and in invasive ductal carcinoma (33/58) relative to other tumor types (2/9), indicating positive associations (Supplementary Table 3).

**Table 3:** Methylation status of TRAIL-R2 promotor region in breast cancer patients

TRAIL-R2 promotor region	Normal	Tumor
ME (0)	25 (37.31%)	36 (53.73%)
UM (1)	42 (62.68%)	31 (46.26%)
Total(*n*)	*n* = 67	*n* = 67

Abbreviations: ME: Methylated; UM: Unmethylated.

**Figure 3:**
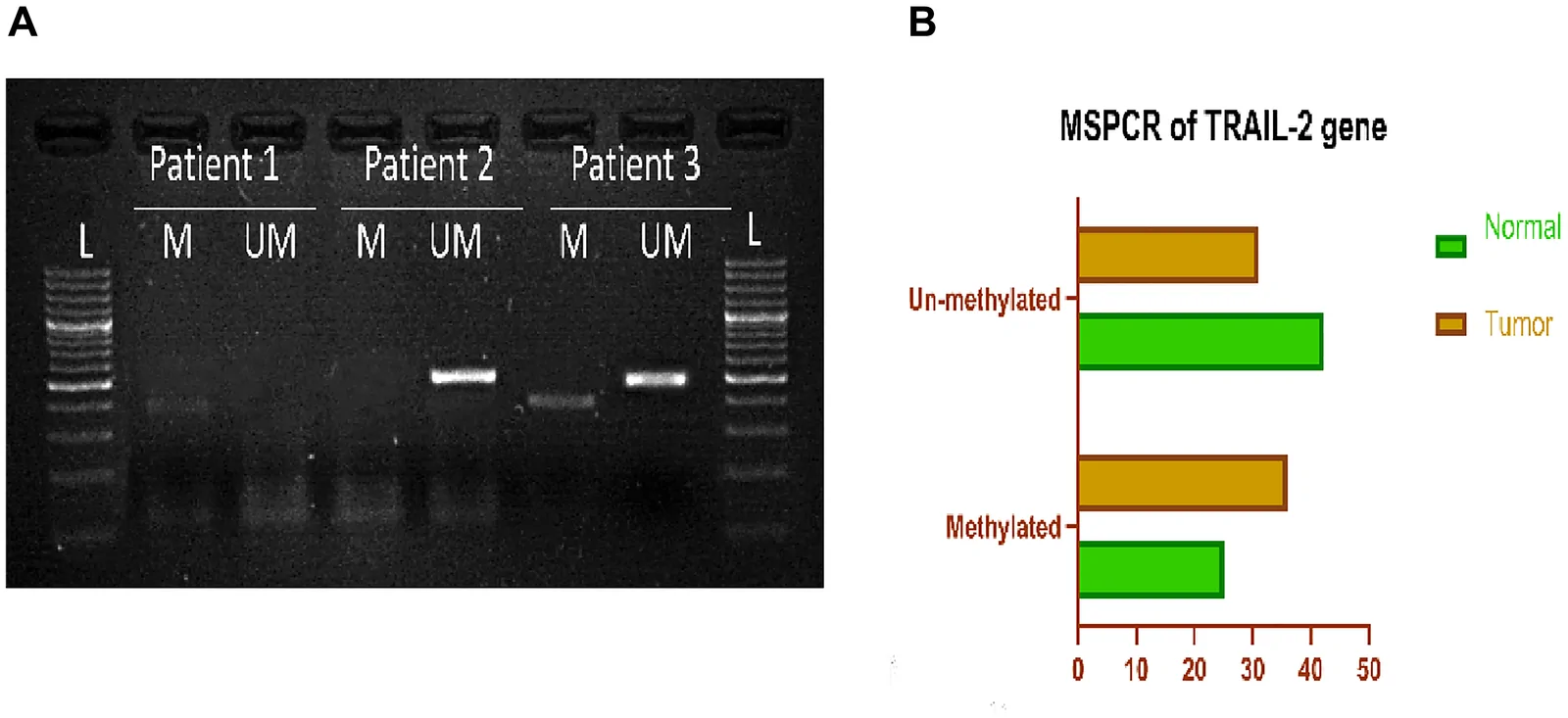
(**A**) Representative gel picture of Methylation-specific PCR analysis of TRAIL-2 gene in Breast cancer patients: DNA methylation was assessed using two specifically designed primers to amplify either methylated DNA (M,386bp), or unmethylated DNA (UM,498bp). (Abbreviations: L: 100 bp DNA ladder; N: normal tissue; T: tumor tissue). (**B**) Graphical representation of Methylation specific PCR of TRAIL-2 gene, showing percentage of methylated and unmethylated samples in breast cancer tissue and adjacent normal tissue.

### Correlation between *TRAIL-R2* methylation and protein expression in stratification by various clinicopathological parameters

There is a significant correlation between protein expression and methylation status when categorized by geographical region, age, menopausal status, breast carcinoma type, laterality, nodal status, necrosis, histological grade, family history, estrogen receptor and progesterone receptor usage, tumor size, and number of pregnancies (Supplementary Table 4).

### Correlation Between gene and protein expression in unmethylated and methylated DNA

To see how gene and protein expression levels were related in unmethylated DNA (UM DNA) areas, Spearman’s rank correlation coefficient was used. The study revealed a modest positive connection (r = 0.444), indicating that increased gene expression levels are often associated with heightened protein expression in these areas (Table 4). The Spearman correlation between gene and protein expression in methylation DNA (ME DNA) regions was lower (r = 0.277), indicating a weaker relationship compared to unmethylated regions (Table 5).

**Table 4:** Spearman correlation between gene and protein expression in unmethylated DNA

	Gene expression	Protein expression
Gene expression	1.000	0.444
Protein expression	0.444	1.000

**Table 5:** Spearman correlation between gene and protein expression in methylated DNA

	Gene expression	Protein expression
Gene expression	1.000	0.277
Protein expression	0.277	1.000

### Kaplan–Meier survival analysis of *TRAIL-R2* protein expression in breast cancer patients

The Kaplan-Meier survival test was conducted on a subset of 30 patients who were evaluated for complete survival and protein expression information. Figure 4 shows the Kaplan-Meier survival curve, which shows how *TRAIL-R2* expression levels affect the overall survival of breast cancer patients. Patients with high *TRAIL-R2* expression, as determined by western blotting, had a significantly better overall survival rate than those with low expression levels (*p* = 0.40).

**Figure 4:**
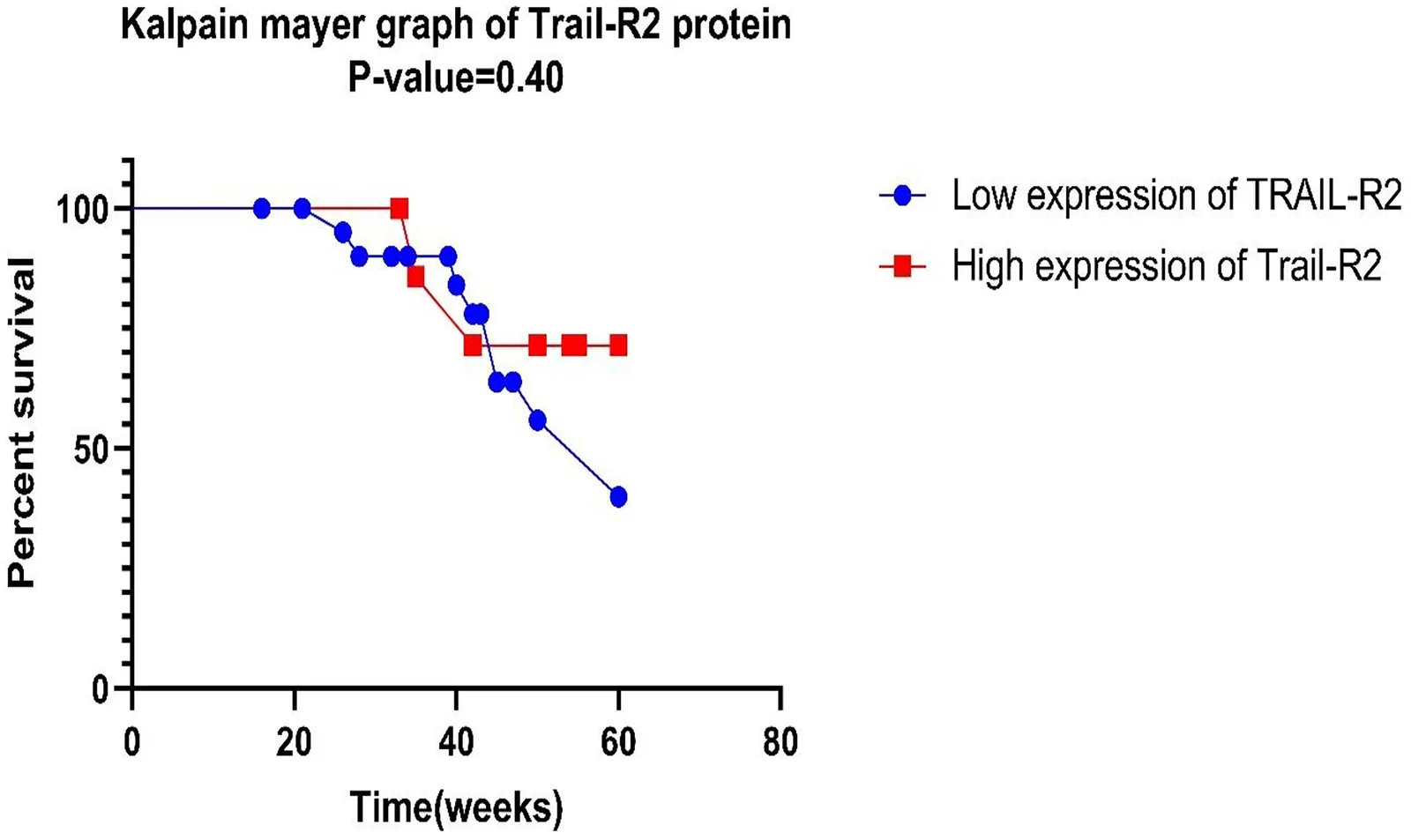
Kaplan-Meier survival study of TRAIL-R2 suggests that red shade signifies high expression of genes, whereas blue denotes low expression. The log-rank test was used to find the *P*-value, and a *P*-value of less than 0.05 was considered statistically significant.

## DISCUSSION

Death receptor 5 *(DR5)*, or *TRAIL-R2*, is a transmembrane receptor that is part of the tumor necrosis factor receptor *(TNFR)* superfamily. It is instrumental in triggering apoptosis via the extrinsic pathway upon activation by its tumor necrosis factor-related apoptosis-inducing ligand *(TRAIL).* The *TNFRSF10B* gene encodes *DR5* and is extensively expressed in diverse organs, including normal cells [27]. The evasion of apoptosis was identified in the late 1980s as a mechanism of cancer genesis and progression and was incorporated in 2000 as one of the original core hallmarks of cancer [28, 29]. The expanding catalogue of genes silenced by hypermethylation of promoter regions allows for the investigation of the inactivation patterns of the *TRAIL-R2* gene in breast cancer tissues [30]. The function of *TRAIL-R2* in apoptosis presents significant promise for the development of successful therapeutic techniques in cancer treatment. We examined the *TRAIL-R2* gene status in Indian female breast cancer cases by rigorously assessing its epigenetic alterations via MSPCR and analysing its expression at the gene level using Real-time PCR and at the protein level through western blotting.

While assessing the *TRAIL-R2* promoter methylation levels in breast cancer samples, we found that tumor samples were highly methylated compared to normal samples. Consistent with our findings, Yong Zhou et al. (2019) reported hypermethylation of the *TRAIL-R2* receptor in tongue carcinoma [31]. Gene silencing via promoter hypermethylation is now seen as a more prevalent occurrence than silence triggered by mutations [32]. Numerous studies have demonstrated the presence of aberrant DNA methylation-either genome-wide or gene-specific—in tumor tissues and corresponding plasma samples from cancer patients, while such alterations are typically absent in healthy controls [33]. Interestingly, Palmisano et al. (2000) reported that in individuals at high risk of lung cancer, such as smokers and uranium miners, abnormal methylation patterns could be detected in sputum samples up to three years before a clinical diagnosis, highlighting its potential as an early biomarker of carcinogenic exposure [34]. In a study on hepatocellular carcinoma, Santella and colleagues identified alterations in the serum methylation profiles of *RASSF1A*, *p16*, and *p15* up to nine years before the disease was diagnosed [35]. Tumor-suppressor genes like *APC* and *ESR1* are often methylated in cancers [36]. *TRAIL-R2* promoter hypermethylation was significantly greater in invasive ductal carcinoma (IDC) than other histological subtypes and was also more in individuals with a history of oral contraceptive use, suggesting a possible correlation between hormonal exposure and epigenetic regulation. A previous study reported a modest inclination toward increased promoter methylation among oral contraceptive users [36]. Also, extended use of oral contraceptives has been definitively associated with an increased risk of developing cervical cancer [37]. We have found higher levels of promoter methylation in IDC compared to other subtypes. Subtype-specific gene regulation is frequently influenced by DNA methylation. One of the previous analyses revealed that genes commonly methylated in Luminal B tumors were generally unmethylated in basal-like tumors, whereas genes methylated in basal-like tumors tended to be unmethylated in Luminal A tumors [38].

Upon analysing the gene expression data, we observed that the expression level of *TRAIL-R2* was downregulated in breast cancer cases. The findings align with a prior study that revealed a decrease in *TRAIL-R2* mRNA expression in breast cancer tissues and BC cell lines relative to normal tissues and nontransformed breast epithelial cell lines [39].

The reduced expression of *TRAIL-R2* mRNA was found to prevail in invasive tumor forms, and was significantly lesser in advanced-stage tumors (TNM stage III–IV) compared to the early-stage tumors. Moreover, the cases that were negative of progesterone receptor showed significantly reduced levels of expression, which indicated an association between the down-regulation of progesterone receptor and aggressive tumor phenotype. These findings align with prior research indicating that the ablation of the TRAIL receptor in mice does not influence primary tumor formation, yet exacerbates lymph node metastasis (LNM) [20], indicating that the TRAIL receptor is crucial for inhibiting tumor spread. Zhuang (2006) similarly revealed that diminished expression of *TRAIL-R2* correlated with metastatic lesions [40]. Our findings align with a prior study demonstrating a substantial connection between *TRAIL-R2* expression and histological grade [41].

While examining protein expression data, we found that most cases showed downregulation of *TRAIL-R2* protein. The downregulation of this protein may be the reason for tumor progression. Studies have reported a marked decrease in *TRAIL-R2* expression in tumors of higher histological grade, indicating a potential inverse relationship between *TRAIL-R2* levels and the aggressiveness of the tumor [42]. A prior study demonstrated that *TRAIL-R2* expression was notably lower in primary tumors exhibiting metastasis, as well as in their associated lymph node metastases, compared to non-metastatic primary tumors. These findings imply a possible link between diminished *TRAIL-R2* expression and increased metastatic capacity in head and neck squamous cell carcinoma [41]. Earlier another study revealed that downregulation of *TRAIL-R2* can promote cancer cell invasion and metastasis by triggering specific signalling pathways. The study further emphasized that lower *TRAIL-R2* expression is linked to a greater number of metastatic lesions in cancers like melanoma and head and neck malignancies [43].

The expression of *TRAIL-R2* protein is significantly lower in postmenopausal patients than it is in premenopausal patients, which supports the idea that the hormonal status of a patient may affect the protein-level regulation of *TRAIL-R2*. Although no study directly addresses *TRAIL-R2* protein expression with menopausal status but they highlight the broader concept that menopausal status can influence hormone receptor expressions in certain cancers. One study reported notable variations in the distribution of breast cancer subtypes between premenopausal and postmenopausal women, indicating that menopausal status may play a role in modulating hormone receptor expression profiles in breast cancer [44]. Another study identified differences in hormone receptor expression between premenopausal and postmenopausal individuals, suggesting that menopausal status may influence the hormonal environment and receptor expression patterns in ovarian cancer [45].

*TRAIL-R2* mediated apoptosis is one of the apoptosis routes that many chemotherapy drugs and radiation treatments use to cause cell death. Because *TRAIL-R2* hypermethylation prevents tumour cells from undergoing apoptosis in response to therapy, it results in therapy resistance. Because *TRAIL-R2* expression is necessary for *TRAIL*-based treatments (TRAIL agonists, recombinant TRAIL), tumours become resistant to *TRAIL*-induced apoptosis when it is silenced [46]. Demethylating drugs, including decitabine, may restore *TRAIL-R2* expression and make tumours more responsive to *TRAIL*-based and traditional treatments, according to certain research [47]. Despite these strong results, the small sample size in our study limits its applicability.

TRAIL-based therapeutic approaches, such as recombinant TRAIL proteins, TRAIL receptor agonistic antibodies, and new approaches using nanoparticulate carriers and cell-based approaches, have been investigated in great detail in order to circumvent cellular resistance in cancer cells, with some approaches already in early-stage clinical development in solid malignancies like breast cancer (e.g., targeting *TRAIL-R2* and approaches that integrate immunotherapeutic agents) [48]. Another important direction of recent work puts new sensitization strategies to the fore that could potentiate TRAIL-induced apoptosis by combinatorial treatments or engineered delivery mechanisms. This emphasizes complex regulation of the TRAIL pathway and points out the necessity for targeted intervention in enhancing clinical efficacy [49]. On the other hand, the role of epigenetics-based prognostic markers, which utilize DNA methylation, has appeared very promising, and several methylated signatures and blood methylated gene targets (GCM2, TMEM240), among others, are being investigated as non-invasive diagnostic predictors that may provide additional information when combined with the expression of proteins such as *TRAIL-R2*, among others [50]. When taken as a whole, these developments in TRAIL regulation, novel treatment approaches, and the identification of methylation biomarkers demonstrate an increasing focus in breast cancer research on combining mechanistic understanding with practical applications.

Furthermore, the study doesn’t investigate how *TRAIL-R2* methylation can interact with other signalling or apoptotic pathways, which could affect the associations seen. The association between *TRAIL-R2* hypermethylation and TNM Staging points to the possibility of using it as a biomarker for the diagnosis and prognosis of breast cancer. Additionally, because DNA methylation is reversible, *TRAIL-R2* can be targeted by epigenetic therapy to restore its pro-apoptotic function. Future research should concentrate on bigger, multi-centre populations to confirm these results and look into the therapeutic potential of demethylating drugs in restoring *TRAIL-R2* expression. Furthermore, mechanistic research is required to investigate how *TRAIL-R2* methylation interacts with other apoptotic regulators in breast cancer.

## MATERIALS AND METHODS

### Collection of human tissue

Between 2020 and 2024, 67 samples of breast tumors and normal breast tissue were donated by the Department of Oncology at the Sher-i-Kashmir Institute of Medical Sciences in Srinagar, Kashmir. This study was endorsed by the Sher-i-Kashmir Institute of Medical Sciences’ Institutional Ethics Committee (IEC) in Srinagar. Until they were required, tissue samples were stored at −80°C after being rapidly frozen in liquid nitrogen. Reports from the pathology department of the Sher-i-Kashmir Institute of Medical Sciences in Soura, Srinagar, provided us with clinicopathological data. Age, menarche and menopausal status, number of pregnancies, use of oral contraceptives, type and side of breast cancer, necrosis, metastasis, tumor size, histological grade, lymph node status, number of lymph nodes, tumor stage, hormone receptor status, HER2 status, triple-negative tumor classification *(ER-, PR-, and HER2-)*, and comorbid status were among the information included. Table 6 displays the results of our investigation attempting to shed light on the relationship between breast cancer cases’ molecular findings and clinical characteristics.

**Table 6:** Study subjects (*n* = 67) and their associated attributes

S. No	Clinicopathological details	Percentage
1.	Gender	*n* = 67 (%)
	Female	*n* = 63 (94.02)
	Male	*n* = 4 (5.97)
2.	Age of subject (in years).	
	Less than or equal to 50	*n* = 43 (64.17)
	More than 50	*n* = 24 (35.82)
3.	Geographical Area	
	Rural	*n* = 52 (77.61)
	Urban	*n* = 15 (22.38)
4.	Status of menopause	
	Pre	*n* = 43 (68.25)
	Post	*n* = 20 (31.74)
5.	Breast Carcinoma type	
	IDC	*n* = 58 (86.56)
	Others	*n* = 9 (13.43)
6.	Breast side	
	Right	*n* = 36 (53.73)
	Left	*n* = 31 (46.26)
7.	Node involvement	
	Negative	*n* = 23 (34.32)
	Positive	*n* = 44 (65.67)
8.	Necrosis	
	Negative	*n* = 7 (10.44)
	Positive	*n* = 60 (89.55)
9.	Metastasis	
	No	*n* = 28 (41.79)
	Yes	*n* = 39 (58.20)
10.	Histological Grade	
	I+II	*n* = 48 (71.64)
	III	*n* = 19 (28.35)
11.	Stage at TNM	
	1 and 2	*n* = 21 (31.34)
	3 and 4	*n* = 46 (68.65)
12.	Family history	
	No	*n* = 53 (79.10)
	Yes	*n* = 14 (20.89)
13.	Estrogen receptor status	
	Negative	*n* = 23 (34.32)
	Positive	*n* = 44 (65.67)
14.	Progesterone receptor status	
	Negative	*n* = 28 (41.79)
	Positive	*n* = 39 (58.20)
15.	Her2 neu receptor status	
	Negative	*n* = 45 (67.16)
	Positive	*n* = 22 (32.83)
16.	Tumor size	
	≤5	*n* = 16 (23.88)
	>5	*n* = 51 (76.11)
17.	Age of menarche	
	≤12	*n* = 19 (30.15)
	>12	*n* = 44 (69.84)
18.	BMI	
	≤25	*n* = 28 (41.79)
	>25	*n* = 39 (58.20)
19.	OCP Use	
	Yes	*n* = 17 (26.98)
	No	*n* = 46 (73.01)
20.	Number of Pregnancies	
	≤3	*n* = 25 (39.68)
	>3	*n* = 38 (60.31)
21.	Comorbidities	
	Nill (0)	*n* = 45 (67.16)
	T2DM (1)	*n* = 5 (7.46)
	COPD (2)	*n* = 1 (1.49)
	HTN (3)	*n* = 8 (11.94)
	Thoracotomy (4)	*n* = 1 (1.49)
	Hypothyroid (5)	*n* = 1 (1.49)
	HTN+others	*n* = 6 (8.95%)

### Selection criteria

This study utilized defined inclusion and exclusion criteria that directed the sampling procedure.

### Inclusion criteria

The inclusion criteria encompassed females aged 23 to 80 with histopathologically confirmed primary breast cancer. The medical record book of Sher-i-Kashmir Institute of Medical Sciences, Soura, Srinagar, kept track of all the patients who took part in the study. The medical records were examined to evaluate the patients’ clinical and pathological data. The local ethical committee’s rules were followed to get informed consent.

### Exclusion criteria

Individuals with a history of major or severe uncontrolled cardiovascular disease were excluded from the trial. Furthermore, patients who have shown a lack of willingness to comply with the protocol were removed.

### DNA extraction

DNA was extracted using the “DNeasy” blood and tissue kit (QIAGEN, GmbH, Germany) from 67 samples of breast cancer tissue and nearby normal, non-cancerous tissue. We verified the quantity and quality of the genomic DNA we recovered using a Nanodrop spectrophotometer (ND1000). The results were then verified using agarose gel electrophoresis.

### Methylation by MS-PCR

The bisulfite conversion of isolated genomic DNA was performed utilising the EZ DNA Methylation-Gold™ Kit. To amplify the altered product, two sets of *TRAIL-2* primers were used: one set with methylation and another set without. We used the Eukaryotic Promoter Database to get the *TRAIL-R2* gene promoter sequence, and then we used Meth Primer to create the primers. The primers employed for detecting methylation in the *TRAIL-R2* promoter region included the sense primer 5′- GAG TAG TGA AAG ATT AGT TCG CGT C -3′ and the antisense primer 5′- CCG AAA CGT TCT ATC CCC G -3′, as well as the sense primer 5′- TGT TTG AGT AGT GAA AGA TTA GTT TGT GTT -3’ and the antisense primer 5′- ACA ACC AAA ACA TTC TAT CCC CA -3′ for identifying unmethylation. The reactions were carried out in a total volume of 25 microliters, with 100 nano grams of bisulfite-treated DNA, 0.2 millimolar of each deoxyribonucleotide (dNTP), 2.5 millimolar of magnesium chloride (MgCl2), 0.4 micromolar of both forward and reverse primers, 0.5 millimolar of GC-rich solution, and one unit of “GENETAQ” DNA Polymerase (PUREGENE, Genetix Biotech Asia Pvt. Ltd.). Amplification was conducted using a Thermal cycler (Mastercycler, Eppendorf), commencing with an initial denaturation phase at 94°C for 30 minutes, succeeded by 40 amplification cycles at 95°C for 30 seconds each. Following amplification, annealing occurred at 51.6°C for methylation and 54.8°C for unmethylation, each for a duration of 30 seconds, succeeded by a 30-second interval at 72°C. The concluding extension occurred at 72°C for 7 minutes. Amplified PCR products were acquired and examined via a Gel Doc system with a 3% agarose gel infused with EtBr, under ultraviolet (UV) illumination (UVP, GelDoc-Ite, Imaging System).

### RNA extraction

TRIzol Reagent (Invitrogen) was used to isolate RNA. According to the manufacturer’s recommendations, the RNA later (Qiagen) kit was used to preserve the breast tissue that had malignant growth and the normal tissues that were next to it.

### Real-time polymerase chain reaction

We utilized a cDNA kit from Promega to synthesize complementary DNA (cDNA) from total RNA. The cDNA was subsequently stored at −20°C for future utilization. The Applied Biosystems Step One real-time PCR device with 48 SYBR Green I Master mix was used to amplify the previously synthesised cDNA in a quantitative polymerase chain reaction (qPCR). We employed the *TRAIL-R2* primers *TRAIL-R2F* (5′-CAT ATG AGT CGG GAC CAA AGA G -3′) and *TRAIL-R2R* (5′-TCC CAG CCT GTC CAT AGA T -3′) to amplify a 113-bp fragment. The *GAPDH* gene was amplified in the identical qPCR experiment and utilized as an internal control. The primers 5′-CAA GAG CAC AAG AGG AAG AGA G -3′ and 5′-CTA CAT GGC AAC TGT GAG GAG -3′ were utilized in the qPCR procedure. This resulted in a product measuring 102 base pairs in length. The PCR cycling parameters were: 5 minutes at 94ºC, followed by 40 cycles of 30 seconds at 94ºC, 30 seconds at 51ºC, and 45 seconds at 72ºC.

The optimization of qRT-PCR was done according to the instructions from the manufacturer. Each experiment included a control sample that did not include any template (no cDNA was present in the PCR). To make sure that each primer pair made a specified product, a melting curve was constructed for each one. We used the 2[-Delta Delta C(T)] approach to figure out the relative quantitation values from the data of just one sample. The housekeeping gene *(GAPDH)* was used to standardize the data for each sample so that it could be compared to normal tissue. This showed fold differences in *TRAIL-R2* expression.

### Western blotting

We took tissue samples, both normal and malignant, out of −80ºC, weighed them, and treated them with trypsin EDTA (Sigma-Aldrich, SLCC7049). After spinning at 13,000 rpm for 45 seconds at 4ºC, the supernatant was taken off and the particle was collected. We washed the pellet twice in PBS (1 ml) at 10,000 rpm for 1 minute each time. The pellet was mixed with 300 microliters of protein lysis buffer, then 10 microliters of PIC, 40 microliters of Naf, and 10 microliters of PMSF. After that, the sample was put on ice for 45 minutes. The sample was spun in a centrifuge at 10,000 rpm for 10 minutes at 4ºC. The supernatant was put into a pre-chilled Eppendorf tube and then kept at −80°C. We used spectrophotometry to measure the proteins, SDS-polyacrylamide gel electrophoresis to separate them, and then we moved them to a nitrocellulose membrane (Amersham TM Protran TM 0.45 μm NC, 10600002, Germany). To stop non-specific binding, the membrane was left in 5% (W/V) Bovine Serum Albumin (BSA) (Sigma, B6917) for 45 minutes. The samples were then put in an incubator at 4°C overnight with the right amounts of anti-DR5 and β-actin primary antibodies (Abcam, Rabbit polyclonal to DR5 antibody ab8416 and Rabbit Polyclonal beta Actin antibody ab8227). After incubation, the membrane was treated with the correct secondary antibody (Goat Anti-Rabbit IgG H and L (HRP) ab205718) (Abcam) for 2 hours and 30 minutes at 4°C. We used an enhanced chemiluminescence (ECL) kit (Millipore, Immobilon R Forte western HRP Substrate, USA) to see the immune-labelled proteins. The β–actin antibody functioned as an internal control.

### Statistical analysis

SPSS, GraphPad Prism, and Microsoft Excel were used to do the statistical analysis. All the experimental assays, including gene expression and protein expression analyses, were carried out in triplicate, with results shown here as mean ± SD. The Chi-square (χ²) test was used for comparison of categorical clinicopathological variables in relation to the status of gene methylation, mRNA expression, and protein expression. The *p*-value of less than 0.05 (*p* < 0.05) was considered as indicating a statistically significant association between variables.

## SUPPLEMENTARY MATERIALS



## Abbreviations

TRAIL-R2: Tumor Necrosis Factor-Related Apoptosis-Inducing Ligand Receptor 2
MSP: Methylation specific PCR
qRT-PCR: Quantitative Real time PCR
OCP: Oral contraceptives
ER: Estrogen receptor
PR: Progesterone receptor
HER-2-neu: Human Epidermal Growth Factor Receptor 2
TNM staging: Tumor, Node, Metastasis classification
BMI: Body Mass Index
T2DM: Type 2 Diabetes Mellitus
COPD: Chronic Obstructive Pulmonary Disease
HTN: hypertension
