# Supplementary material for: TRAIL-R2 in the shadows: Epigenetic silencing and clinical implications in breast cancer
Source: Oncotarget. 2026 Jun 9;17:316–28. doi: 10.18632/oncotarget.28891 (PMC13637834; doi:10.18632/oncotarget.28891)
Supplement: Supplementary file 1 [file oncotarget-17-28891-s001.pdf]

TRAIL-R2 in the shadows: Epigenetic silencing and clinical implications in breast cancer

SUPPLEMENTARY MATERIALS

Supplementary Table 1: Correlation study of TRAIL-R2 mRNA expression levels with clinical parameters of breast cancer case

Characteristics	Total (*n* = 67)	TRAIL-R2 mRNA expression relative to GAPDH (Mean ± SD)	*P*-value	Chi-squared
Gender	*n* = 67			
Female	*n* = 63	0.42 ± 0.3	0.22	1.44
Male	*n* = 4	0.29 ± 0.05		
Age				
≤50	*n* = 43	0.37 ± 0.21	0.44	0.57
>50	*n* = 24	0.49 ± 0.5		
Geographical Area				
Rural	*n* = 52	0.43 ± 0.4	0.22	1.48
urban	*n* = 15	0.3 ± 0.2		
Status of menopause				
Pre	*n* = 43	0.36 ± 0.2	0.32	0.95
Post	*n* = 20	0.55 ± 0.6		
Breast Carcinoma type				
IDC	*n* = 58	0.3 ± 0.1	0.0002***	13.72
Others	*n* = 9	0.9 ± 0.8		
Breast side				
Right	*n* = 36	0.41 ± 0.2	0.19	1.65
left	*n* = 31	0.41 ± 0.5		
Node involvement				
Negative	*n* = 23	0.5 ± 0.6	0.85	0.03
positive	*n* = 44	0.36 ± 0.1		
Necrosis				
Negative	*n* = 7	0.4 ± 0.2	0.21	1.54
Positive	*n* = 60	0.4 ± 0.4		
Metastasis				
No	*n* = 28	0.48 ± 0.5	0.40	0.68
Yes	*n* = 39	0.36 ± 0.1		
Histological Grade				
I+II	*n* = 48	0.45 ± 0.4	0.07	3.08
III	*n* = 19	0.31 ± 0.2		
Stage at TNM				
1 and 2	*n* = 21	0.55 ± 0.6	0.04*	3.98
3 and 4	*n* = 46	0.35 ± 0.1		

Family history				
No	*n* = 53	0.4 ± 0.4	0.12	2.30
Yes	*n* = 14	0.44 ± 0.2		
Estrogen receptor status				
Negative	*n* = 23	0.46 ± 0.2	0.10	2.68
Positive	*n* = 44	0.39 ± 0.4		
Progesterone receptor status				
Negative	*n* = 28	0.54 ± 0.5	0.002**	9.36
positive	*n* = 39	0.32 ± 0.1		
Her2 neu receptor status				
Negative	*n* = 45	0.44 ± 0.4	0.80	0.06
Positive	*n* = 22	0.35 ± 0.1		
Tumor size				
≤5	*n* = 16	0.52 ± 0.7	0.84	0.03
>5	*n* = 51	0.38 ± 0.2		
Age of menarche				
≤12	*n* = 19	0.42 ± 0.2	0.24	1.34
>12	*n* = 44	0.42 ± 0.4		
BMI				
≤25	*n* = 28	0.36 ± 0.22	0.77	0.08
>25	*n* = 39	0.45 ± 0.4		
OCP Use				
yes	*n* = 17	0.44 ± 0.2	0.47	0.51
no	*n* = 46	0.41 ± 0.4		
Number of Pregnancies				
≤3	*n* = 25	0.31 ± 0.2	0.31	1.02
>3	*n* = 38	0.45 ± 0.4		
Comorbidities				
Nill	*n* = 45	0.43 ± 0.45	0.93	1.81
T2DM	*n* = 5	0.33 ± 0.16		
COPD (2)	*n* = 1	0.288 ± 0.00		
HTN (3)	*n* = 8	0.38 ± 0.18		
Thoracotomy (4)	*n* = 1	0.188 ± 0.00		
Hypothyroid (5)	*n* = 1	0.33 ± 0.00		
HTN+others	*n* = 6	0.94 ± 0.23		

*$p < 0.05$ = statistically significant, **$p < 0.01$ = highly significant, ***$p < 0.001$ = very highly significant.

Supplementary Table 2: Correlation of TRAIL-R2 protein expression level with clinical parameters of breast cancer patients

Characteristics	Total Cases (*n* = 67)	TRAIL-R2 Absent/(low)	TRAIL-R2 Present	*P*-Value	Chi-Squared	Odds ratio
Gender	*n* = 67					
Female	*n* = 63	47 (74.60)	16 (25.39)	0.98	0.0003	0.97
Male	*n* = 4	3 (75)	01 (25)			(0.071–6,977)

Age						
≤50	*n* = 43	30 (69.76)	13 (30.23)	0.22	1.49	0.46
> 50	*n* = 24	20 (83.33)	4 (16.66)			(0.149–1.589)
Geographical Area						
Rural	*n* = 52	41 (78.84)	11 (21.15)	0.13	2.18	2.48
urban	*n* = 15	9 (60)	6 (40)			(0.703–7.967)
Status of menopause						
Pre	*n* = 43	29 (67.44)	14 (32.55)	0.05	3.66	0.23
Post	*n* = 20	18 (90)	2 (10)			(0.048–0.966)
Breast Carcinoma type						
IDC	*n* = 58	41 (70.68)	17 (29.31)	0.66	0.19	0.68
Others	*n* = 9	07 (77.77)	02 (22.22)			(0.134–3.088)
Breast side						
Right	*n* = 36	25 (69.44)	11 (30.55)	0.29	1.10	0.54
left	*n* = 31	25 (80.64)	06 (19.35)			(0.164–1.671)
Node involvement						
Negative	*n* = 23	18 (78.26)	5 (21.73)	0.62	0.24	1.35
positive	*n* = 44	32 (72.72)	12 (27.27)			(0.413–4.008)
Necrosis						
Negative	*n* = 7	06 (85.71)	01 (14.28)	0.47	0.50	2.18
Positive	*n* = 60	44 (73.33)	16 (26.66)			(0.296–26.46)
Metastasis						
No	*n* = 28	20 (71.42)	08 (28.57)	0.61	0.25	0.750
Yes	*n* = 39	30 (76.92)	09 (23.07)			(0.237–2.174)
Histological Grade						
I+II	*n* = 48	35 (72.91)	13 (27.08)	0.60	0.26	0.71 (0.227–2.601)
III	*n* = 19	15 (78.94)	04 (21.05)			
Stage at TNM						
1 and 2	*n* = 21	16 (76.19)	05 (23.80)	0.84	0.03	1.12
3 and 4	*n* = 46	34 (73.91)	12 (26.08)			(0.338–3.397)
Family history						
No	*n* = 53	38 (71.69)	15 (28.30)	0.28	1.14	0.42 (0.086–1.870)
Yes	*n* = 14	12 (85.71)	2 (14.28)			
Estrogen receptor status						
Negative	*n* = 23	19 (82.60)	04 (17.39)	0.27	1.17	1.99
Positive	*n* = 44	31 (70.45)	13 (29.54)			(0.574–6.185)
Progesterone receptor status						
Negative	*n* = 28	24 (85.71)	04 (14.28)	0.07	3.12	3.00
positive	*n* = 39	26 (66.66)	13 (33.33)			(0.883–9.207)
Her2 neu receptor status						

Negative	*n* = 45	36 (80%)	09 (20%)	0.28	1.13	1.86
Positive	*n* = 22	15 (68.18)	07 (31.81)			(0.607–5.516)
Tumor size						
≤5	*n* = 16	13 (81.25)	03 (18.75)	0.48	0.48	1.64
>5	*n* = 51	37 (72.54)	14 (27.45)			(0.453–6.011)
Age of menarche						
≤12	*n* = 19	15 (78.94)	04 (21.05)	0.60	0.27	1.40
>12	*n* = 44	32 (72.72)	12 (27.27)			(0.373–4.487)
BMI						
≤25	*n* = 28	20 (71.42)	08 (28.57)	0.61	0.25	0.750
>25	*n* =	30 (76.92)	09 (23.07)			(0.237–2.174)
OCP Use						
yes	*n* = 17	13 (76.47)	04 (23.52)	0.83	0.04	1.14
no	*n* = 46	34 (73.91)	12 (26.08)			(0.341–3.707)
Number of Pregnancies						
≤3	*n* = 25	18 (72)	07 (28)	0.37	0.80	0.580
>3	*n* = 38	31 (81.57)	07 (18.42)			(0.178–1.885)
Comorbidities						
Nil	*n* = 45	23 (51.11)	22 (48.88)	0.62	4.37	
T2DM	*n* = 5	02 (40)	03 (60)			
COPD	*n* = 1	00 (0)	01 (100)			
HTN	*n* = 8	05 (62.5)	03 (37.5)			
Thoracotomy	*n* = 1	00 (0)	01 (100)			
Hypothyroid	*n* = 1	01 (100)	00 (0)			
HTN+OTHERS	*n* = 6	02 (33.33)	04 (66.66)			

Supplementary Table 3: Correlation study of TRAIL-R2 Promoter Methylation status with clinical parameters of Breast Cancer Patients

Characteristics	Total Cases (*n* = 67)	Methylated	Unmethylated	*P*-Value	Chi-Squared	Odds ratio (95% CI)
Gender	*n* = 67					
Female	*n* = 63	35 (55.55%)	28 (44.44%)	0.23	1.413	3.750
Male	*n* = 4	01 (25%)	03 (75%)			(0.524–49.89)
Age						
≤50	*n* = 43	23 (53.48)	20 (46.51)	0.95	0.002	0.973 (0.381–2.791)
>50	*n* = 24	13 (54.16)	11 (45.83)			
Geographical Area						
Rural	*n* = 52	28 (53.84)	24 (46.15)	0.97	0.001	1.021
urban	*n* = 15	08 (53.33)	07 (46.66)			(0.337–2.954)
Status of menopause						
Pre	*n* = 43	23 (53.48)	20 (46.51)	0.62	0.234	0.766 (0.270–2.151)
Post	*n* = 20	12 (60)	08 (40)			

Breast Carcinoma type						
IDC	*n* = 58	33 (56.89)	25 (43.10)	0.05	3.75	4.62
Others	*n* = 9	02 (22.22)	07 (77.77)			(0.849–23.01)
Breast side						
Right	*n* = 36	20 (55.55)	16 (44.44)	0.74	0.104	1.172
left	*n* = 31	16 (51.61)	15 (48.38)			(0.443–3.130)
Node involvement						
Negative	*n* = 23	12 (52.17)	11 (47.82)	0.853	0.034	0.909
positive	*n* = 44	24 (54.54)	20 (45.45)			(0.351–2.347)
Necrosis						
Negative	*n* = 7	03 (42.85)	04 (57.14)	0.542	0.372	0.613
Positive	*n* = 60	33 (55)	27 (45)			(0.145–2.466)
Metastasis						
No	*n* = 28	14 (50)	14 (50)	0.604	0.269	0.772
Yes	*n* = 39	22 (56.41)	17 (43.58)			(0.281–2.087)
Histological Grade						
I+II	*n* = 48	28 (58.33)	20 (41.66)	0.22	1.442	1.92
III	*n* = 19	08 (42.10)	11 (57.89)			(0.681–5.382)
Stage at TNM						
1 and 2	*n* = 21	10 (47.61)	11 (52.38)	0.498	0.460	0.699
3 and 4	*n* = 46	26 (56.52)	20 (43.47)			(0.254–1.869)
Family history						
No	*n* = 53	28 (52.83)	25 (47.16)	0.44	0.58	0.622
Yes	*n* = 14	09 (64.28)	05 (35.71)			(0.2.0–2.015)
Estrogen receptor status						
Negative	*n* = 23	16 (69.56)	07 (30.43)	0.060	3.532	2.74 (0.910–7.39)
Positive	*n* = 44	20 (45.45)	24 (54.54)			
Progesterone receptor status						
Negative	*n* = 28	15 (53.57)	13 (46.42)	0.982	0.00	0.989
positive	*n* = 39	21 (53.84)	18 (46.15)			(0.366–2.712)
Her2 neu receptor status						
Negative	*n* = 45	26 (57.77)	19 (42.22)	0.342	0.903	1.64
Positive	*n* = 22	10 (45.45)	12 (54.54)			(0.613–4.469)
Tumor size						
≤5	*n* = 16	10 (62.5)	06 (37.5)	0.425	0.650	1.60
>5	*n* = 51	26 (50.98)	25 (49.01)			(0.500–5.429)
Age of menarche						
≤12	*n* = 19	11 (57.89)	08 (42.10)	0.806	0.060	1.14
>12	*n* = 44	24 (54.54)	20 (45.45)			(0.395–3.257)
BMI						
≤25	*n* = 28	15 (53.57)	13 (46.42)	0.982	0.0004	0.98
>25	*n* = 39	21 (53.84)	18 (46.15)			(0.366–2.712)

OCP Use						
yes	$n = 17$	13 (76.47)	04 (23.52)	0.042*	4.125	3.54 (1.021–10.93)
no	$n = 46$	22 (47.82)	24 (52.17)			
Number of Pregnancies						
≤3	$n = 25$	16 (64)	09 (36)	0.490	0.474	1.43
>3	$n =$	21 (55.26)	17 (44.73)			(0.483–4.123)
Comorbidities						
Nill (0)	$n = 45$	25 (55.55)	20 (44.44)	0.68	3.90	–
T2DM (1)	$n = 5$	03 (60)	02 (40)			
COPD (2)	$n = 1$	01 (100)	00 (0)			
HTN (3)	$n = 8$	03 (37.5)	05 (62.5)			
Thoracotomy (4)	$n = 1$	00 (0)	01 (100)			
Hypothyroid (5)	$n = 1$	01 (100)	00 (0)			
HTN+others	$n = 6$	03 (50)	03 (50)			

*$p < 0.05$ = statistically significant.

Supplementary Table 4: Correlation analysis between TRAIL-R2 methylation and TRAIL-R2 protein expression in stratification by various clinical characteristics of Breast cancer patients.

Clinical Characteristics	Total (N)	TRAIL-R2 methylation status	TRAIL-R2 Expression		*P*-value	Chi-Squared	Odds ratio
Gender			Absent	Present			
Female (63)	35	M	27	08	0.219	1.50	2.14
		UM	11	17			0.61–7.26
Male (04)	01	M	00	01	0.50	0.44	0.00
		UM	01	02			0.00–27.00
Age							
≤50(43)	23	M	17	06	0.05	3.74	3.46
		UM	09	11			1.03–10.88
> 50(24)	13	M	10	03	0.015*	5.91	8.88
		UM	03	08			1.35–43.28
Geographical Area							
Rural (52)	28	M	26	02	<0.0001****	33.83	91.00
		UM	03	21			13.21–453.1
Urban (15)	08	M	07	01	0.06	3.34	9.33
		UM	03	04			0.66–127.0
Status of menopause							
Pre (43)	23	M	14	06	0.0080**	7.03	5.83
		UM	06	15			1.61–21.87
Post (20)	12	M	10	02	0.0113*	6.41	11.67
		UM	03	07			1.35–68.83
Breast Carcinoma type							

IDC (58)	33	M	28	05	<0.0001****	19.21	14.40
		UM	7	18			3.90–52.00
Others (9)	03	M	03	00	0.017*	5.62	–
		UM	01	05			
Breast side							
Right (36)	20	M	16	04	0.024*	5.06	5.14
		UM	07	09			1.11–18.32
Left (31)	16	M	12	04	0.007**	7.24	8.25
		UM	04	11			1.46–39.70
Node involvement							
Negative (23)	11	M	08	03	0.05	3.56	5.33
		UM	04	08			0.85–25.04
Positive (44)	24	M	20	04	0.001**	10.75	9.28
		UM	07	13			2.38–31.13
Necrosis							
Negative (7)	03	M	02	01	0.27	1.21	6.0
		UM	01	03			0.28–111.1
Positive (60)	30	M	25	05	0.0002***	13.61	8.63
		UM	11	19			2.66–26.52
Metastasis							
No (28)	14	M	11	03	0.22	1.47	2.75
		UM	08	06			0.52–12.01
Yes (39)	22	M	11	11	0.85	0.03	0.88
		UM	09	08			0.24–3.14
Histological Grade							
I+II (48)	28	M	21	07	0.014*	5.97	4.50
		UM	08	12			1.2–14.4
III (19)	08	M	07	01	0.0094**	6.73	18.67
		UM	03	08			1.93–230.0
Stage at TNM							
1 and 2(21)	10	M	07	03	0.12	2.37	4.08
		UM	04	07			0.57–19.95
3 and 4(46)	26	M	19	07	0.05	3.74	3.31
		UM	09	11			0.88–12.45
Family history							
No (53)	28	M	25	03	<0.0001****	26.80	35.00
		UM	05	21			7.92–130.6
Yes (14)	08	M	07	01	0.07	3.25	10.50
		UM	02	03			0.86–151.1
Estrogen receptor status							
Negative (23)	16	M	13	03	0.06	3.39	5.77
		UM	03	04			0.87–31.13
Positive (44)	20	M	18	02	<0.0001****	20.92	34.20
		UM	05	19			5.82–166.4

Progesterone receptor status							
Negative (28)	15	M	11	04	0.50	0.44	1.71
		UM	08	05			0.30–7.04
Positive (39)	20	M	17	03	<0.0001****	18.68	30.22
		UM	03	16			4.91–130.3
Her2 neu receptor status							
Negative (45)	26	M	17	09	0.12	2.40	2.59
		UM	08	11			0.70–8.99
Positive (22)	10	M	06	04	0.74	0.10	0.750
		UM	08	04			0.14–3.80
Tumor size							
≤5 (16)	10	M	07	03	0.88	0.019	1.16
		UM	04	02			0.15–7.87
>5 (51)	26	M	18	08	0.03*	4.39	3.37
		UM	10	15			1.07–10.37
Age of menarche							
≤12 (19)	11	M	07	04	0.25	1.26	2.91
		UM	03	05			0.51–15.14
>12(44)	24	M	16	08	0.03*	4.38	3.71
		UM	07	13			0.98–11.92
BMI							
≤25 (28)	15	M	09	06	0.93	0.0069	0.93
		UM	08	05			0.18–4.31
>25(39)	21	M	14	07	0.16	1.94	2.50
		UM	08	10			0.70–8.18
OCP Use							
Yes (17)	13	M	08	05	0.20	1.63	4.80
		UM	01	03			0.52–68.81
No (46)	22	M	17	05	0.001**	10.65	8.25
		UM	07	17			2.02–31.90
Number of Pregnancies							
≤3(25)	14	M	10	04	0.07	3.07	4.37
		UM	04	07			0.80–23.97
>3(38)	21	M	16	05	0.0003***	12.88	14.93
		UM	03	14			2.92–59.07
Comorbidities							
Nill (45)	25	M	14	11	0.28	1.13	1.90
		UM	08	12			(0.53–6.17)
T2DM (5)	03	M	02	01	0.13	2.22	Infinity
		UM	00	02			(0.33–infinity)
COPD (1)	01	M	00	01	–	–	–
		UM	00	00			

HTN (8)	03	M	02	01	0.18	1.74	8.00 (0.39–139.1)
		UM	01	04			
Thoracotomy (1)	00	M	00	00	–	–	–
		UM	00	01			
Hypothyroid (1)	01	M	01	00	–	–	–
		UM	00	00			
HTN+others (6)	03	M	02	01	0.08	3.00	Infinity (0.53–infinity)
		UM	00	03			

*$p < 0.05$ = statistically significant, **$p < 0.01$ = highly significant, ***$p < 0.001$ = very highly significant, ****$p < 0.0001$ = extremely significant
